# Analysis of Naturally Occurring Steroid Hormones in Infant Formulas by HPLC-MS/MS and Contribution to Dietary Intake

**DOI:** 10.3390/foods4040605

**Published:** 2015-10-22

**Authors:** Rocío Barreiro, Patricia Regal, Mónica Díaz-Bao, Cristina A. Fente, Alberto Cepeda

**Affiliations:** Department of Analytical Chemistry, Nutrition and Bromatology, Faculty of Veterinary Science, University of Santiago de Compostela, 27002 Lugo, Spain; E-Mails: rocio.barreiro@usc.es (R.B.); monica.diaz@usc.es (M.D.-B.); cristina.fente@usc.es (C.A.F.); alberto.cepeda@usc.es (A.C.)

**Keywords:** infant formula, steroid hormone, estrogen, progestogen, androgen, breast milk, bovine milk, daily intake, LC-MS/MS

## Abstract

Milk is a natural fluid and as such contains small amounts of naturally occurring steroids. Human milk is recommended as the optimal source of nutrients for infants and young children, and it has been associated to several short- and long-term benefits. For this reason, its composition is used as a reference for designing infant formulas. However, the available information on the hormonal levels of these dairy products is scarce, and it is usually limited to estradiol and estrone. In the present study, six natural sex hormones (pregnenolone, progesterone, estrone, dehydroepiandrosterone, testosterone and androstenedione) have been extracted from sixteen milk-based infant formulas and analyzed with liquid chromatography tandem mass spectrometry (HPLC-MS/MS). The purpose of this research was to quantify natural steroid hormones in various infant formulas, to provide food and nutrition practitioners with information to estimate intakes in children. In addition, data found in the literature was used for comparison. The findings suggest that there are certain similarities between bovine milk and dairy products for infants. Furthermore, the detected levels were in general lower than those observed in human milk and/or colostrum. The reported results represent a valuable addition to the current knowledge on natural hormone content of infant foods.

## 1. Introduction

Human milk is traditionally recommended as the optimal source of nutrients for the infant. All its bioactive molecules and functional factors are considered necessary for good postnatal health and proper development of newborns [1]. Additionally, it has been associated to several short- and long-term benefits for the child. For these reasons, its composition is used as a reference in the manufacture of infant formulas, industrially produced human milk substitutes intended for infant consumption. The nutritional and bioactive profiles of breast milk are highly variable among and within individuals over time, and as such they are impossible to mimic in infant formulas [2]. The composition of these dairy products is relatively constant, and it is usually adapted to the specific needs of the child in a specific age range. Energy, protein, lipids and even amino acids content are controlled by the manufacturers in order to match breastfeeding performance. However, certain compounds such as hormones and live cells are not added, monitored or modified in infant formulas, and the existing information on its presence in breast milk is also scarce.

The presence of endogenous steroid hormones in milk and other animal-based foods is unavoidable as they naturally occur in animals. Actually, there is concern among some consumers about the safety and potential adverse health effects of these hormones that are present in food [3]. With regard to the use of steroids in farm animals, the Directive 96/22/EC regulated hormonal treatments in the European Union, in order to protect consumers’ health [4]. However, natural steroid hormones cannot be avoided in food of animal origin, since they are part of the animal metabolism [5,6,7]. In addition, their concentrations vary with the kind of food, species, gender, age and physiological stage of the animal [5,8,9]. Bovine milk also naturally contains considerable amounts of hormones, and it is of particular concern, mainly due to the practices of modern dairy farms [9,10,11]. Most infant formulas available in the market and other products that are also frequently consumed by children are based on modified cow`s milk. Baby foods have special functions in infant diets because they are major source of nutrients and a unique source of food during the first months of life. These products are specifically covered by Commission Directive 2006/141/EC [12], amended by the Commission Directive 2013/46/EU [13]. Dairy preparations for infants and children can be considered an important source of natural hormones, even though the existing information about their concentration in these products is very limited [14,15,16,17]. Therefore, more of these hormonal compounds should be quantified in different types of products to obtain an estimation of their consumption and to compare them with the levels found in human breast milk [18,19,20].

To date, there have been no reports comparing the hormonal levels in baby milk with the existing data on the hormonal levels in human breast milk and in bovine milk. Additionally, there is an obvious lack of data in the literature about formula composition. This is what makes it difficult to determine whether potential exposures are large when compared with acceptable daily intakes or with the natural levels present in mothers’ milk. The objectives of this study were to perform an evaluation of the presence of six primary steroid hormones (two progestogens, one estrogen and three androgens) in milk-based infant preparations and to compare their hormonal composition with the existing data. In addition, this study provides the food and nutrition experts with information to estimate potential consumption of hormones in babies and young children. The selected liquid chromatography tandem mass spectrometry (HPLC-MS/MS) methodology was previously validated for determining six hormones in bovine milk, in accordance with the Commission Decision 2002/657/EC [9,21]. Eleven commercial infant and follow-on formulas and three types of growing-up milk have been analyzed, in order to evaluate the levels of this six sex steroids. One sample of lactose-free milk (intended for children 3–12 years) and one sample of milk for children between three and twelve years were also analyzed. The obtained results were compared with data found in the literature for bovine milk, human milk and infant formulas. The maximum daily intakes for sex hormones in infants (aged 0–12 months), young children (aged 1–3 years) and children aged 3–12 years old were also estimated.

## 2. Experimental Section

### 2.1. Reagents and Standard Solutions

Experimental materials included pregnenolone (P_5_) [5-pregnen-3ß-ol-20-one], progesterone (P_4_) [4-pregnen-3,20-dione], dehydroepiandrosterone (DHEA) [5-androsten-3β-ol-17-one], androstenedione (A) [4-androsten-3,17-dione], testosterone (T) [4-androsten-17β-ol-3-one] and estrone (E_1_) [1,3,5(10)-estratien-3-ol-17-one], provided by Steraloids Ltd. (Croydon, UK) and deuterated analogues of steroids (P_5_-d_4_, E_1_-d_2_ and DHEA-d_2_) from CDN Isotopes (Quebec, QC, Canada). Activated charcoal, formic acid, methyl tert-butylether (MTBE), methanol and acetonitrile were supplied by Merck (Darmstadt, Germany). Hydroxylamine solution (99.999%, 50 wt.% in H_2_O), ascorbic acid and β-glucuronidase from *Helix pomatia* were supplied by Sigma-Aldrich (St Louis, MO, USA). Baker analyzed LC-MS water from J.T. Baker (Deventer, The Netherlands) was used in the analysis.

Standard solutions containing 1.0 mg·mL^−1^ of each hormone were prepared separately in methanol with 0.1% ascorbic acid to prevent oxidation and stored in the dark at −20 °C. Working standard solutions containing all assayed steroids were prepared by dilution, using methanol with 0.1% ascorbic acid at concentrations of 10, 15, 20, 30, 40, 50, 75 and 100 ng·mL^−1^ and stored at 5 °C. An internal standard (IS) solution was prepared with a mixture of the deuterated analogues (P_5_-d_4_, E_1_-d_2_ and DHEA-d_2_) at 1 ng·mL^−1^ in acetonitrile 0.1% ascorbic acid.

### 2.2. Samples

**Figure 1:**
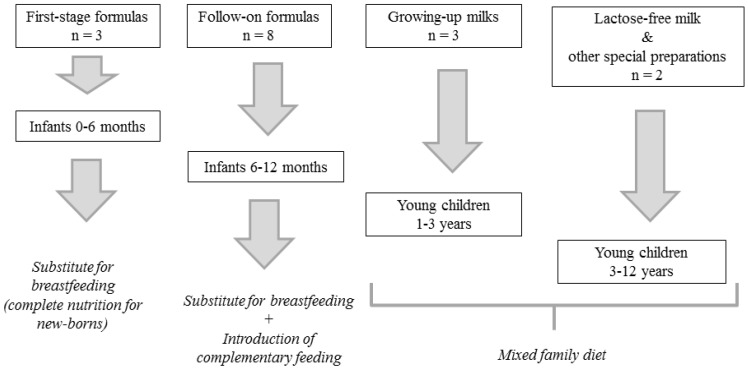
Classification of the different milk-based preparations for infants and children analyzed in this study (formulas and milk).

Fourteen commercial bovine milk-based formulas were randomly collected between different brands existing in the Spanish market in 2012, including three first-stage infant formulas (intended for babies from 0 to 6 months of age), eight follow-on infant formulas (intended for babies from six months on) and three types of growing-up milk (intended for young children, from one to three years). Samples were stored in the dark at −20 °C until analysis. The powdered formulas were reconstituted in water to obtain liquid formulas, following the manufacturer’s instructions. One sample of lactose-free milk and one sample of milk for children between three and twelve years were also analyzed. See Figure 1 for more details on sample classification.

### 2.3. Sample Preparation

**Figure 2:**
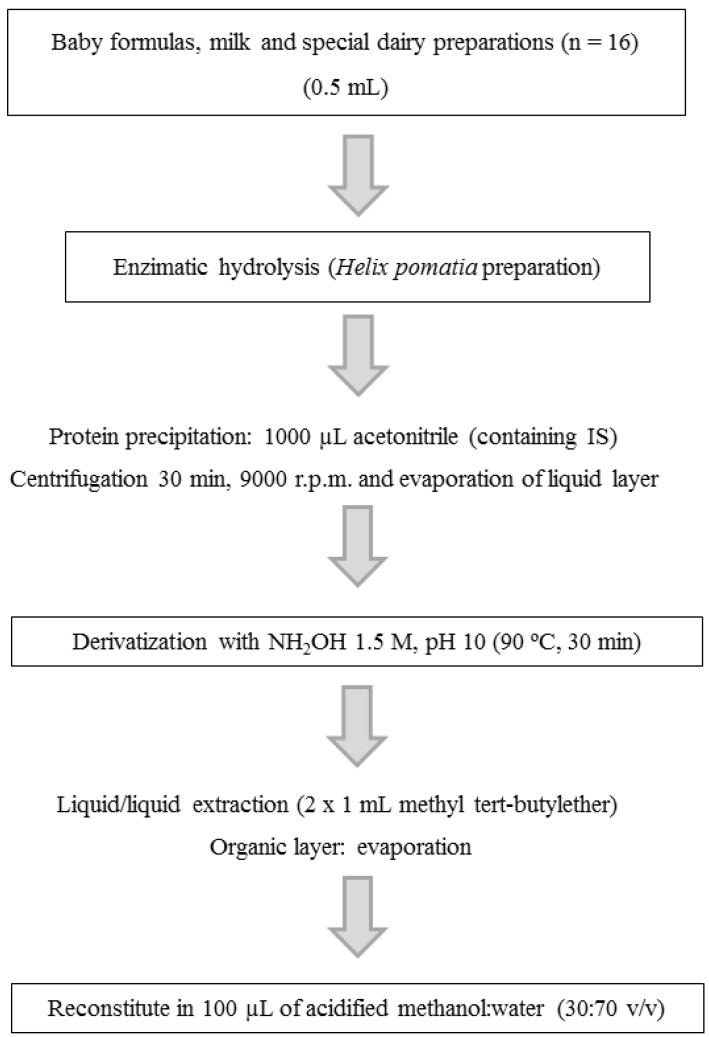
Overview of the sample preparation used for HPLC-MS/MS analysis of milk samples.

As steroids are naturally present in milk, activated charcoal was added to a pool of all the milk samples in order to remove hormones from milk-based infant preparations, and thus a blank matrix was obtained [9]. Spiked samples (infant formulas spiked with pregnenolone, progesterone, dehydroepiandrosterone, androstenedione, testosterone and estrone at a known concentration) and blank samples for the matrix-matched calibration curve were prepared using the obtained blank milk and the working standard solutions. Matrix-matched and standard solution calibration curves were prepared in a range of 20–200 ng·dL^−1^, by addition of 10 μL of working standard solutions at different concentrations. A schematic overview of sample preparation is presented in Figure 2. Before the extraction procedure, hydrolysis was performed with β-glucuronidase in order to determine the sum of free and conjugated steroids, as previously reported [9]. Briefly, samples of each reconstituted infant formula and calibrators (500 μL) were placed in a 2 mL tube containing 1000 μL of the IS solution (1 ng of deuterium-labeled hormones). Tubes were capped, vortexed and centrifuged (16,100× g, 25 min) using an Eppendorf Centrifuge 5415D (Hamburg, Germany). The acetonitrile layer was separated from the precipitate and evaporated under a nitrogen stream at 37 °C. The assayed natural steroids were derivatized with 300 μL of 1.5 M hydroxylamine solution (pH 10) at 90 °C for 30 min. Oxime derivatives were extracted twice with MTBE and, after evaporation of the organic layer under a nitrogen stream, the residue was re-dissolved in 100 μL of acidified methanol-water (30:70 *v*/*v*) and immediately analyzed by HPLC-MS/MS.

### 2.4. HPLC-MS/MS Measurement of Hormonal Levels

The liquid chromatographic separation was achieved using an Agilent 1100 series HPLC quaternary gradient pump system (Agilent Technologies, MN, USA), and a Synergi Fusion-RP 100A (100 × 2 mm, 2.5 μm plus Security Guard cartridge) from Phenomenex (Torrance, CA, USA). Elution solvents were water (A) and methanol (B) containing 0.1% formic acid. Mobile phase composition (A:B; *v*/*v*) was 90:10 at 0 min and held for 3 min, from 90:10 to 20:80 in 5 min, from 20:80 to 50:50 in 5 min, from 50:50 to initial conditions 90:10 in 8 min and then held for 2 min for column re-equilibration. Flow rate was 0.2 mL·min^−1^, and the injected volume was 65 μL. Mass spectrometric measurement was achieved using an Q Trap 2000 triple quadrupole instrument from AB Sciex (Toronto, ON, Canada), operating with Ion Source Turbo Spray in positive mode. Two diagnostic signals (multiple reaction monitoring or MRM transitions) were monitored for each target compound, using the optimized source and MS parameters [9].

Data were collected using a Dell Optiplex GX400 workstation and processed with the Analyst 1.4.1 software package (MDS SCIEX). The concentrations of the analytes in real samples were interpolated from matrix-matched calibration curves, which were constructed by calculating the area ratios (analyte peak area/IS peak area) *versus* analyte concentration in ng·dL^−1^. The calibration graph can be described by the equation *y = mx + b*.

The HPLC-MS/MS method was previously validated according to Commission Decision 2002/657/EC. Because there was no certified reference material available in our laboratory, this Decision offers a means of determining the accuracy of corrected recovery for spiked samples. Spiked samples were prepared using blank milk fortified with the working standard solutions. The recovery study was performed with six replicates of the first three spiking levels of the calibration curves, at 20, 40 and 60 ng·dL^−1^. The recovery was corrected for losses during sample preparation via the aid of a deuterated internal standard. The obtained mean recoveries were 96.9% for P_5_, 92.6% for P_4_, 101.5% for E_1_, 102.6% for A, 101.0% for T and 96.2% for DHEA [9]. For all steroids, the recovery was in compliance with the European criteria to be between 70% and 110%. The obtained decision limits (CCα) and detection capabilities (CCβ) for P_5_, P_4_, DHEA, A, T and E_1_ in a range of 5.15–9.04 and 8.77–15.41 ng·dL^−1^, respectively [9]. When an analyte was not detected (*i.e.*, below its CCα), its value was expressed as the CCα. When an analyte was detected but not quantifiable accurately (*i.e.*, below its CCβ), its value was expressed as the CCβ [22]. Each sample was analyzed three times (on three consecutive days) and the hormonal concentration for each dairy preparation was expressed as the mean value of the three measurements, in ng per dL of reconstituted formula/milk. Descriptive parameters (mean and standard deviation) and other calculations were obtained using the Microsoft Excel software package version 2010 (Microsoft Corp., Redmond, WA, USA).

### 2.5. Calculation of Daily Intakes

The theoretical daily intakes of hormones were calculated on the basis of the mean concentrations obtained for each hormone in each milk-based formula/product. The daily consumption of these products (dL of milk) was estimated using hypothetical scenarios, based on other published reports and on intake recommendations from Nestle Baby website [23,24]. In this study, the mean consumption for each formula was estimated at 9 dL per day for infants up to six months, 7 dL for infants between six and twelve months, and 5 dL for young children from one to twelve years. These estimations were used to obtain the theoretical intakes of natural steroid hormones (in μg of hormones—androgens, estrogens, progestogens—per day) when the baby or the child is consuming the products analyzed in this study. For each dairy preparation, its hormonal concentration was multiplied by the amount of milk ingested daily by the infant in each age range.

## 3. Results and Discussion

The intake of natural steroid hormones in babies and children is difficult to estimate by food and nutrition practitioners due to the lack of information on formula composition. To date, very few studies have reported the levels of these natural compounds in formulas and human breast milk. Moreover, in most cases the data in the literature is limited to estrone (E_1_) and/or estradiol (E_2_) [15,16,18,19,20]. In this study, six naturally occurring steroid hormones have been determined and quantified in milk and milk formulas intended for babies and children. Additionally, some data found in literature for bovine milk, infant formulas and human breast milk was collected and used for comparison. With the help of the existing data and these new results, nutrition and food experts could estimate the intake of hormonal compounds through milk ingestion. In addition, a comparison with their natural presence in human breast milk is now possible, especially in the case of infant formula.

### 3.1. Samples

Food products specifically designed for infants (aged 0–12 months) are available on the market in all European countries. The composition of infant formulas is regulated by Directives 2006/141/EC and its recent amendment, the Commission Directive 2013/46/EU [12,13]. The Directive 2006/141/EC establishes the requirements for the composition and labeling of infant formula and follow-on formula. The annexes of the Directive give criteria for the composition (protein, carbohydrate, fat, mineral substances, vitamins and certain other ingredients) of infant formulas and follow-on formulas including, where necessary, minimum and maximum levels. The present Directive also encompasses the specific rules on the presence of pesticides residues and it requires that baby food contains no detectable levels of pesticide residues and prohibits the use of certain very toxic pesticides in the production of infant and follow-on products. According to the legislation, “infant formulas” or “first-stage formulas” are used during the first months of life until the appropriate introduction of complementary feeding that is when “follow-on formulas” are used, usually at six months of age.

Baby formula milk is usually made from cow’s milk that has been modified to make it suitable for babies and to match the composition of breast milk as closely as possible. First-stage infant formulas offer complete nutrition for newborns (from birth up to six months) and they’re easy to digest. In this study, these formulas were considered as unique source of nutrients for the child, as well as unique source of exogenous hormones (Figure 1). However, a combination of breast milk and formula feeding is also possible, and in this case the total intake of hormones would be different. On the other hand, follow-on formulas are designed for older babies (from six months of age up to one year) who have started eating solid foods. In this respect, it is important to point out that, in the case of the latter formulas as well as the rest of dairy products, nutrition and food experts should take into account other sources of hormones to estimate the total daily intake. From six months on, there is a possibility of ingestion of other food of animal origin that may contribute to the total hormonal intake of the child (Figure 1).

### 3.2. Analytical Methodology

**Figure 3:**
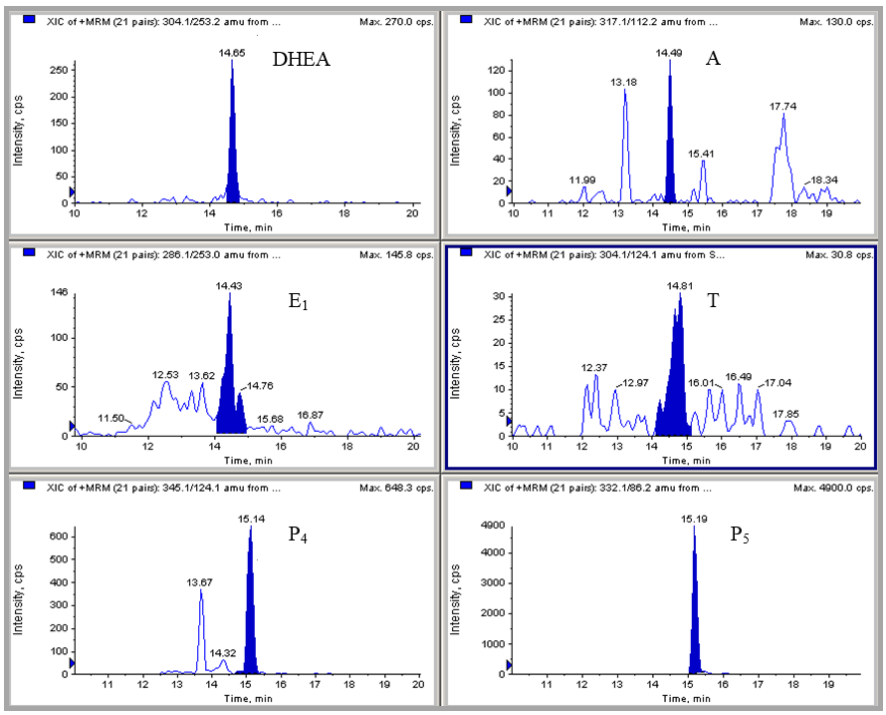
LC-MS/MS chromatogram of a first-stage infant formula, showing the presence of all the assayed hormones: pregnenolone (P_5_), progesterone (P_4_), estrone (E_1_), dehydroepiandrosterone (DHEA), testosterone (T), and androstenedione (A).

An HPLC-MS/MS method previously validated in the laboratory for the analysis of hormones in milk was used to analyze sixteen dairy formulas and commercial preparations [9]. The samples included in this study were three first stage formulas and eight follow-on formulas for infants, and three types of growing-up milk, one lactose-free milk and one special preparation for young children, all from different brands commercialized in Spain (Table 1). First-stage infant formulas are intended for babies from zero to six months of age, follow-on for babies from six months on, and three types of growing-up milk for young children from one to three years. The last two samples (lactose-free milk, special dairy preparation for young children) are intended for children between three and twelve years. The selected analytical method enables the quantification of six main natural hormones (pregnenolone (P_5_), progesterone (P_4_), estrone (E_1_), testosterone (T), androstenedione (A) and dehydroepiandrosterone (DHEA)) which have a ketone group in their structure for hydroxylamine derivatization. Regarding this procedure, oximation is considered to be one of the most interesting reactions for derivatization as it offers an increased steroid ionization when using electrospray ionization (ESI) sources. Due to the affinity of NH_2_OH for ketone groups, the analysis of E_2_ could not be included in the study. Estradiol does not have any ketone group in its structure to react with hydroxylamine. Even though E_1_ is less biologically potent than E_2_, it is an important estrogen in bovine milk. It has been observed that the mammary gland preferentially concentrates E_1_, since its content is markedly higher than that of E_2_ [25]. Based on this fact, the analysis of E_1_ in formulas and preparations obtained from bovine milk was considered as important as E_2_ determination. A chromatogram obtained during the HPLC-MS/MS analysis of one of the first stage formulas (sample 1) is shown in Figure 3.

**Table 1:** Mean concentrations of six natural steroid hormones in different baby formulas and dairy products (ng·dL^−1^); estimated daily intake of androgens, progestogens and estrone, calculated using the estimated consumption (dL per day) of each product.

Dairy Product (dL per Day)		% Fat	Hormonal Levels (ng·dL^−1^) *						Estimated Daily Intake (μg)		
			A	T	DHEA	E_1_	P_4_	P_5_	A + T + DHEA	E_1_	P_5_ + P_4_
First-stage formula (9 dL)	1	4.0	20.9 ± 5.7	10.7 ± 2.9	28.9 ± 0.8	19.3 ± 13.3	91.4 ± 31.7	614.4 ± 117.1	0.5	0.2	6.4
	2	3.2	11.2 ± 3.7	7.3 ± 0.0	10.2 ± 3.3	8.8 ± 0.0	10.5 ± 3.9	157.4 ± 185.6	0.3	0.1	1.5
	3	3.5	11.2 ± 3.7	7.3 ± 0.0	27.9 ± 28.5	20.4 ± 20.2	12.8 ± 5.6	48.4 ± 41.4	0.4	0.2	0.6
Follow-on formula (7 dL)	1	3.0	13.3 ± 3.7	9.0 ± 2.9	10.2 ± 3.3	40.8 ± 50.1	12.8 ± 7.5	50.5 ± 9.1	0.2	0.3	0.4
	2	3.1	13.5 ± 3.9	7.3 ± 0.0	13.1 ± 8.3	25.3 ± 14.5	31.8 ± 31.1	76.0 ± 86.3	0.2	0.2	0.8
	3	2.8	11.2 ± 3.7	7.3± 0.0	16.5 ± 14.2	22.7 ± 18.9	14.5 ± 7.0	45.3 ± 0.8	0.2	0.2	0.4
	4	3.2	14.3 ± 4.8	11.1 ± 3.3	8.3 ± 0.0	35.5 ± 41.0	20.8 ± 1.7	67.2 ± 4.0	0.2	0.2	0.6
	5	2.9	15.2 ± 6.0	7.3 ± 0.0	8.3 ± 0.0	8.8 ± 0.0	16.2 ± 8.7	36.1 ± 26.6	0.2	0.1	0.4
	6	3.2	12.2 ± 4.5	7.3 ± 0.0	11.2 ± 4.1	24.9 ± 14.0	22.6 ± 0.1	74.2 ± 21.8	0.2	0.2	0.7
	7	2.8	9.0 ±0.0	7.3 ± 0.0	8.3 ± 0.0	8.8 ± 0.0	25.1 ± 0.8	30.1 ± 1.5	0.2	0.1	0.4
	8	3.0	12.2 ± 4.5	7.3 ± 0.0	8.3 ± 0.0	11.9 ± 4.4	34.8 ± 5.9	319.7 ± 212.4	0.2	0.1	2.5
Growing-up (5 dL)	1	2.7	11.2 ± 3.7	10.7 ± 5.8	11.4 ± 5.5	21.9 ± 17.5	54.6 ± 16.4	69.0 ± 12.1	0.2	0.1	0.6
	2	3.0	11.2 ± 3.8	7.3 ± 0.0	8.3 ± 0.0	20.7 ± 20.6	21.1 ± 9.0	88.3 ± 97.2	0.1	0.1	0.5
	3	3.2	17.2 ± 2.0	7.3 ± 0.0	14.1 ± 7.8	8.8 ± 0.0	10.9 ± 0.0	5.2 ± 0.0	0.2	0.0	0.1
Milk 3–12 years(5 dL)	1	2.8	16.9 ± 2.1	7.3 ± 0.0	27.9 ± 7.8	11.9 ± 4.4	102.8 ± 45.1	87.9 ± 9.8	0.3	0.1	1.0
Lactose-free milk (5 dL)	1	1.6	21.1 ± 1.3	7.3 ± 0.0	8.3 ± 0.0	8.8 ± 0.0	13.9 ± 6.6	8.8 ± 0.0	0.2	0.0	0.1

* Results were given as means ± SD from triplicate estimations.

For all the analyzed samples, the sum of conjugated and free hormones was determined (enzymatic hydrolysis). This approach was preferred in order to simplify the analysis and also because of the relatively high proportion of conjugated species of steroid hormones existing in milk [25,26,27]. The conjugated forms are not biologically active, but they can be transformed into their free active form in the human gut. To perform the analysis, liquid samples were prepared from powdered formulas according to the manufacturer’s directions, and all samples were analyzed three times (on three consecutive days). The hormonal levels of these dairy preparations were calculated as the mean value of those three analyses.

### 3.3. Measurement of Natural Steroid Hormones in Dairy Preparations for Infants

The concentrations (mean ± SD) observed for the natural hormones in the dairy preparations included in this study were summarized in Table 1, as well as their declared fat content. Some authors have associated the concentration of this type of compounds with the fat content of milk [25,26]. Actually, the obtained results allow for visualization of this association in the case of the first sample, especially for pregnenolone. Other researchers have reported the levels of some naturally occurring hormones in different types of bovine milk (whole, skim, raw, from pregnant cows, *etc.*), using different analytical methods (HPLC-MS/MS, immunoassays) [6,9,19,20,26,28,29,30,31]. So far, there has been limited research on the hormonal levels of infant formulas prepared using bovine milk [15,19] or on the hormone content of human milk [18,19,20]. Some of the data obtained in this study and a summary of the results found in the literature have been included in Table 2, in order to facilitate comparisons between different formulas and various types of milk (human, bovine, commercial). Infant formulas and breast milk and/or colostrum are highlighted in bold.

This study revealed that, in general, there are similarities between bovine milk and baby milk (Table 2). These formulas typically start with cow milk as a base so this finding is not surprising. In most formula, estrone levels were slightly higher to those found in infant formula from China, but these authors only analyzed the free fraction [15]. However, the levels of estradiol and estrone found in these Chinese formulas resulted clearly higher than in the infant formula analyzed by Azzouz *et al.* in 2011 [19]. There is little data on the levels of pregnenolone in milk. The present results were quite high and similar to the levels found in raw milk from pregnant animals [9]. As for progesterone, only a few of the analyzed samples were in the range of milk produced by pregnant cows. The rest had lower levels. In general, the hormonal composition of the different infant formulas included in Table 2 reflects a high variability. Considering that samples came from individual brands (and obviously different milk batches), this variability was already expected.

**Table 2:** Mean concentrations of five natural steroid hormones, determined in different infant formulas, milk for children, bovine milk and human breast milk.

Analysis	Sample		Hormonal Levels (ng·dL^−1^)					Ref.
			T	A	P_4_	E_1_	17β-E_2_	
Total content (deconjugated)	First-stage formula	Sample 1	**10.7**	**20.9**	**91.4**	**19.3**	–	Results in this article
	First-stage formula	Sample 2	**7.3**	**11.2**	**10.5**	**8.8**	–	
	First-stage formula	Sample 3	**7.3**	**11.2**	**12.8**	**20.4**	–	
	Follow-on formula	Sample 1	**9.0**	**13.3**	**12.8**	**40.8**	–	
	Follow-on formula	Sample 2	**7.3**	**13.5**	**31.8**	**25.3**	–	
	Follow-on formula	Sample 3	**7.3**	**11.2**	**14.5**	**22.7**	–	
	Follow-on formula	Sample 4	**11.1**	**14.3**	**20.8**	**35.5**	–	
	Follow-on formula	Sample 5	**7.3**	**15.2**	**16.2**	**8.8**	–	
	Follow-on formula	Sample 6	**7.3**	**12.2**	**22.6**	**24.9**	–	
	Follow-on formula	Sample 7	**7.3**	**9.0**	**25.1**	**8.8**	–	
	Follow-on formula	Sample 8	**7.3**	**12.2**	**34.8**	**11.9**	–	
	Growing-up formula	Sample 1	**10.7**	**11.2**	**54.6**	**21.9**	–	
	Growing-up formula	Sample 2	**7.3**	**11.2**	**21.1**	**20.7**	–	
	Growing-up formula	Sample 3	**7.3**	**17.2**	**10.9**	**8.8**	–	
	Milk 3–12 years	Sample 1	**7.3**	**16.9**	**102.8**	**11.9**	–	
	Lactose-free	Sample 1	**7.3**	**21.1**	**13.9**	**8.8**	–	
Free hormones only	Infant formula milk powder	Sample 1	–	–	–	**3.6**	**1.9**	[15]
		Sample 2	–	–	–	**5.0**	**2.5**	
		Sample 3	–	–	–	**14.6**	**3.3**	
		Sample 4	–	–	–	**14.4**	**3.5**	
		Sample 5	–	–	–	**2.7**	**3.7**	
		Sample 6	–	–	–	**2.7**	**3.8**	
Free and sulfate conjugates	Skim fraction	from raw milk	–	–	–	0.1	–	[26]
	Raw milk whole	50 cows	–	–	–	0.2	–	
	Fat fraction	from raw milk	–	–	–	2.8	–	
	Whole milk	pasteurized	–	–	–	1.0	–	
	Raw bovine milk	173 cows	–	–	–	0.7	–	
Total content (deconjugated)	Bovine raw milk	pregnant	10.3	36.7	82.4	14	–	[9]
	Bovine raw milk	non-pregnant	8	10.9	8.2	8.8	–	
Total content (deconjugated)	Bovine colostrum	1 day	10	18	646	130.0	30	[20]
	Bovine milk	26–30 days	10	10	213	–	–	
	Human colostrum	1 day	**25**	**131**	**239**	**27.0**	–	
	Human colostrum	3 days	**19**	**98**	**101**	**17.0**	–	
Free hormones only	Raw bovine milk	Sample 1	–	–	–	7.5	310	[19]
		Sample 2	–	–	–	8.0	320	
		Sample 3	–	–	–	2.5	9	
	Whole	Sample 1	–	–	–	3.5	110	
		Sample 2	–	–	–	13.0	120	
	Human milk	Sample 1	–	–	–	**5.5**	**54**	
		Sample 2	–	–	–	**17.0**	**49**	
	Powdered infant milk	Sample 1	–	–	–	**<0.01**	**<0.12**	
Total content (deconjugated)	Human milk	concentration range (*n* = 4)	–	–	–	–	**790 to 1850**	[18]

On the other hand, there are no reference levels or legal limits for hormones in baby food. Also, no declared hormone values were found on the label of each product. Consequently, no legal conclusions can be drawn regarding its hormonal content, or in terms of food safety. As human milk is considered to be a perfect food for children, with adequate amount of nutrients and other biological compounds, its hormonal content could be used as a reference by food and nutrition experts. In the latter respect, the values found in the literature for human milk and/or colostrum were in all cases higher than the levels found in the formulas of this study, except for progesterone and estrone in some of the samples. The concentrations obtained for progesterone were rather variable, and only two of the baby products were in the range of human colostrum. The rest of the samples had lower levels of P_4_. The estrone levels observed in the milk analyzed in this study were also variable, showing concentrations in the range of human colostrum and mature milk [19,20], or even higher than one-day colostrum in two cases. It is, nevertheless, important to note that human colostrum may present higher values of these natural hormones. In 2011, Xu *et al.* obtained results which evidence that hormonal levels in human colostrum follow a decreasing trend from day one postpartum to day three [20]. Breast milk is highly variable among and within individuals over time, so the differences between the three human studies reflected in Table 2 are normal [18,19,20]. As for estradiol, that was not measured in this research, the results recently obtained by Chen *et al.* for Chinese formulas were lower than the estradiol levels found in human milk in two different studies, one measuring only free hormones and the other the total content of estradiol [15,18,19]. Therefore, it may be stated that the amount of steroid hormones ingested through bovine milk and/or formulas is similar or lower than the amount that the infant/child would ingest through breastfeeding. Unfortunately, there was no data available to check the similarities in pregnenolone and DHEA content between formulas and breast milk.

### 3.4. Hormonal Intake Calculation

Newborns are exposed to maternal hormones through breast-feeding. Accordingly, maternal milk is exclusive source of exogenous hormones during the first months of life of the child. When complementary feeding is introduced, approximately at six months of age, the intake calculation would imply many kinds of foods of animal origin. It will then be easier to calculate the total intake during the first months of life, when milk is the sole source of hormones. If breastfeeding is not possible or it cannot be exclusive feeding for the infant, formulas are used. For research purposes and to estimate the intake of natural hormones, the first-stage formulas (for infants from zero to six months old) were accounted as complete substitutes of breast-feeding and unique source of exogenous hormones. However, follow-on formulas, growing-up formulas and the rest of dairy preparations for children are not and cannot be considered the unique source of nutrients. Further considerations should be taken when assessing their contribution to the daily intake of hormones. It is important to note that complimentary feeding of animal origin would also contribute to the total daily intake of these compounds.

A theoretical daily intake of each group of hormones (androgens, progestogens and estrone) was calculated on the basis of estimated daily ingestions of milk for infants weighing 8 kg (six-month-old), 10 kg (one-year-old), 15 kg (three-year-old) or 40 kg (12-year-old). The estimated ingestion for infants during the first six months of life was 900 mL, from six to twelve months 700 mL and from one to twelve years 500 mL, per day. The intake results were in a range of 0.13–0.54 μg/day for androgens, 0.04–0.29 μg/day for estrogens (estrone) and 0.08–6.35 μg/day for progestogens, with an average of 0.41, 0.15 and 2.8 μg/day, for infants 0–6 months old, 0.22, 0.16, 0.77 μg/day for infants from 6 to12 months old, 0.16, 0.09, 0.41 μg/day for children from 1 to 3 years old, and 0.22, 0.05, 0.53 μg/day for children three to twelve years old, respectively. Even though the first-stage formulas resulted in higher daily intakes, there are no other sources of hormones to be considered. The rest of formulas and preparations gave lower values but they may not be the only source of hormones that the child is consuming in the diet. Table 1 shows the estimated daily ingestions of the assayed steroid hormones (in μg) through the exclusive consumption of each preparation.

The theoretical food intake may be compared to the acceptable daily intake (ADI) established by the Joint Food and Agriculture Organization of the United Nations (FAO) and World Health Organization (WHO) Expert Committee on Food Additives (JECFA) and the permitted increase exposure established by the US Food and Drug Administration (FDA), for progesterone, 17β-testosterone and 17β-estradiol [32,33]. The estimated body weights for children six, 12, 36 months and 12 years old were eight, 10, 15 and 40 kg, respectively, and as such were used for ADI calculations. The recommendations of JECFA and FDA for P_4_, T and βE_2_ are shown in Table 3. As these are the only official recommendations existing nowadays for hormone ingestion, nutrition and food experts can use them as a reference. All the results obtained in this study were below the ADI levels for the corresponding age range. The permitted increase exposure established by the US FDA is clearly lower than ADI, and for this reason almost all samples were above 100% of the permitted exposure for estrone, except the three last samples. For androgens and progestogens, 100% of the FDA permitted exposure was only reached through first-stage formulas, and one follow-on formula in the case of progestogens.

**Table 3:** Maximum intake recommendations (per day) for three main natural steroid hormones in different groups of population.

CRITERIA	PARAMETER	P_4_	T	βE_2_
JECFA, 1999 [32]	ADI ^a^	0–30 μg·kg^−1^ bw	0–2 μg·kg^−1^ bw	0–50 ng·kg^−1^ bw
	ADI adult 60 kg	1.8 mg·day^−1^	120 μg·day^−1^	3 μg·day^−1^
	ADI child 0.5 year old (8 kg)	0.24 mg·day^−1^	16 μg·day^−1^	0.4 μg·day^−1^
	ADI child 1 year old (10 kg)	0.3 mg·day^−1^	20 μg·day^−1^	0.5 μg·day^−1^
	ADI child 3 years old (15 kg)	0.45 mg·day^−1^	30 μg·day^−1^	0.75 μg·day^−1^
	ADI child 12 years old (40 kg)	1.2 mg·day^−1^	80 μg·day^−1^	2 μg day^−1^
	NOEL ^b^ (LOEL ^c^ for P_4_)	3.3 mg·kg^−1^ bw·day^−1^	1.7 mg·kg^−1^ bw·day^−1^	5 μg·kg^−1^ bw·day^−1^
FDA, 2006 [33]	DAILY PRODUCTION	150 μg	32 μg	6 μg
	permitted increase exposure	1.5 μg	0.32 μg	0.06 μg

Notes: ^a^ ADI: acceptable daily intake; ^b^ NOEL: non-observed effect level; ^c^ LOEL: lowest-observed effect level.

Manufacturers modify milk for babies by adjusting carbohydrate, protein, and fat levels, and adding vitamins and minerals. However, no procedures are performed to control or even adjust the hormone content of these preparations, even though this study suggests that it may be necessary.

## 4. Conclusions

Natural hormones cannot be avoided in food of animal origin, since they are part of animal metabolism. Actually, human milk is also a source of hormones for the newborn and the breast-fed child. The present study provides data on the occurrence of hormones in some commercial dairy products for infants and children marketed in Spain. The obtained hormonal levels were, in general, higher for progestogens and estrogens (estrone) than for androgens. In Europe, no regulation has been published yet with regard to the hormonal content of food of animal origin, not even for infant formulas or baby food. Consequently, no legal assessment could be addressed with regard to maximum permitted levels. The official recommendations established by the JECFA and the US FDA were set for specific compounds and not for a sum of similar compounds, so potency factors for different androgens, estrogens and progestogens should be considered. Furthermore, a possible ingestion of hormones through other food of animal origin has not been considered in this study, but it may contribute to the total hormone intake.

The estimated intakes of hormones are a valuable tool for future nutritional and endocrine considerations. This study revealed that there were similarities between the hormone levels found in dairy products for infants and children and the hormone content of bovine milk. However, the levels of hormones found in human milk were, in general, higher than in the rest of milk.
